# Supplementary figures and images for: T-complex protein 1 subunit zeta-2 (CCT6B) deficiency induces murine teratospermia
Source: PeerJ. 2021 Jun 1;9:e11545. doi: 10.7717/peerj.11545 (PMC8176918; doi:10.7717/peerj.11545)

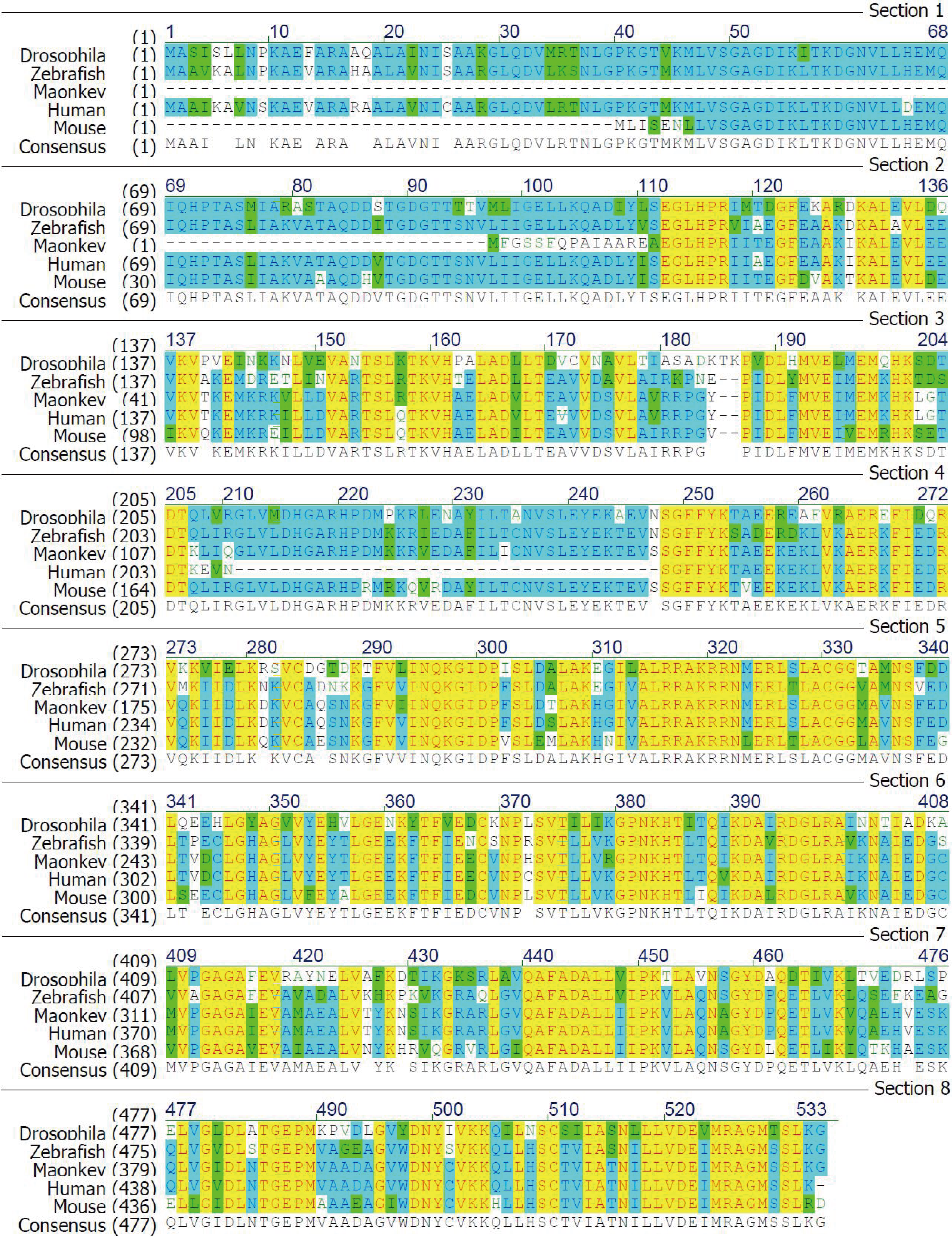

Supplement: Supplemental Information 1 — Sequence alignment of CCT6B proteins from drosophila, zebrafish, monkey, human and mouse. [file peerj-09-11545-s001.png]

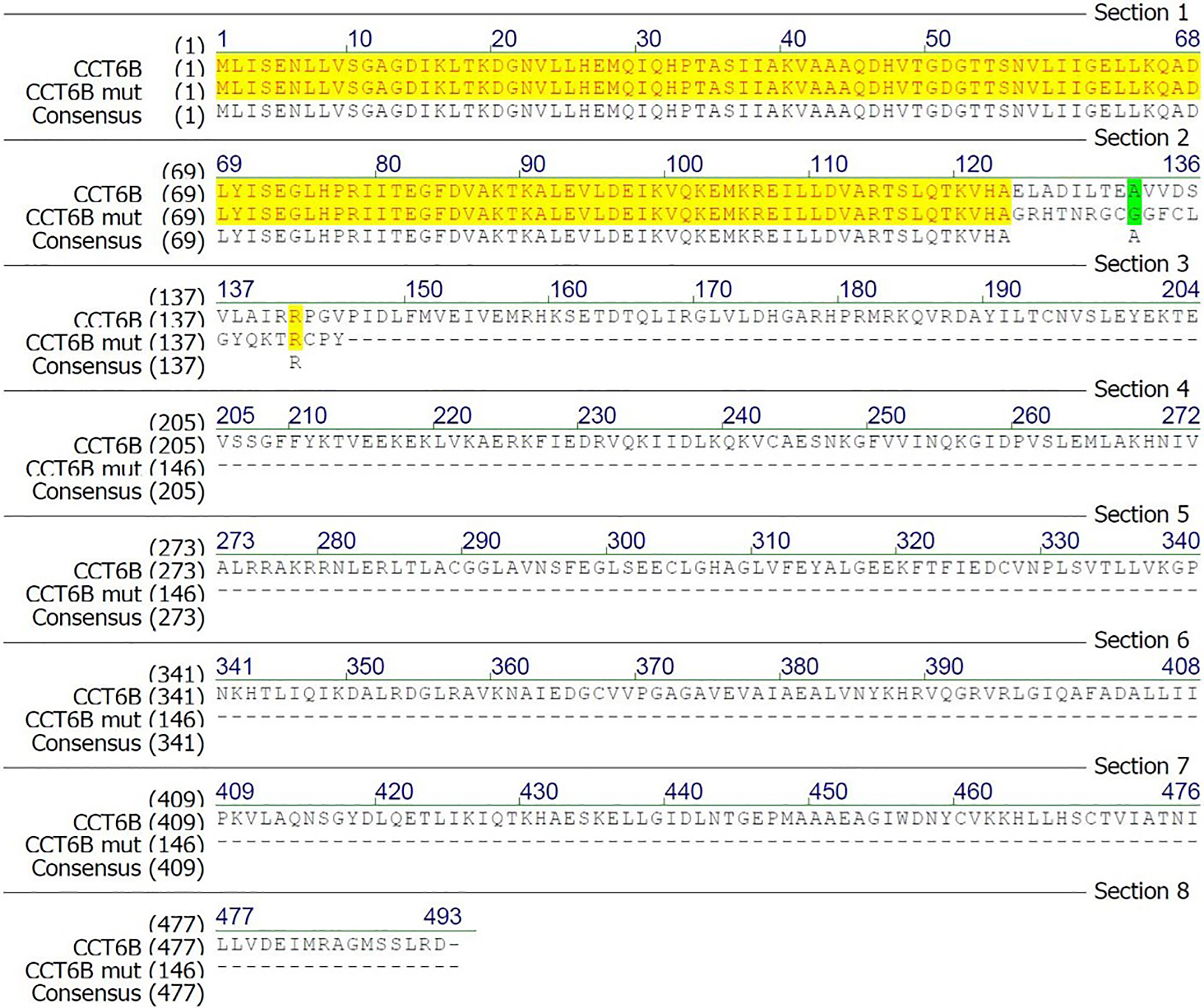

Supplement: Supplemental Information 2 — The sequences of CCT6B and CCT6B mutated proteins were compared, revealing that the mutation led to a frameshift and produced a premature termination codon. [file peerj-09-11545-s002.jpg]

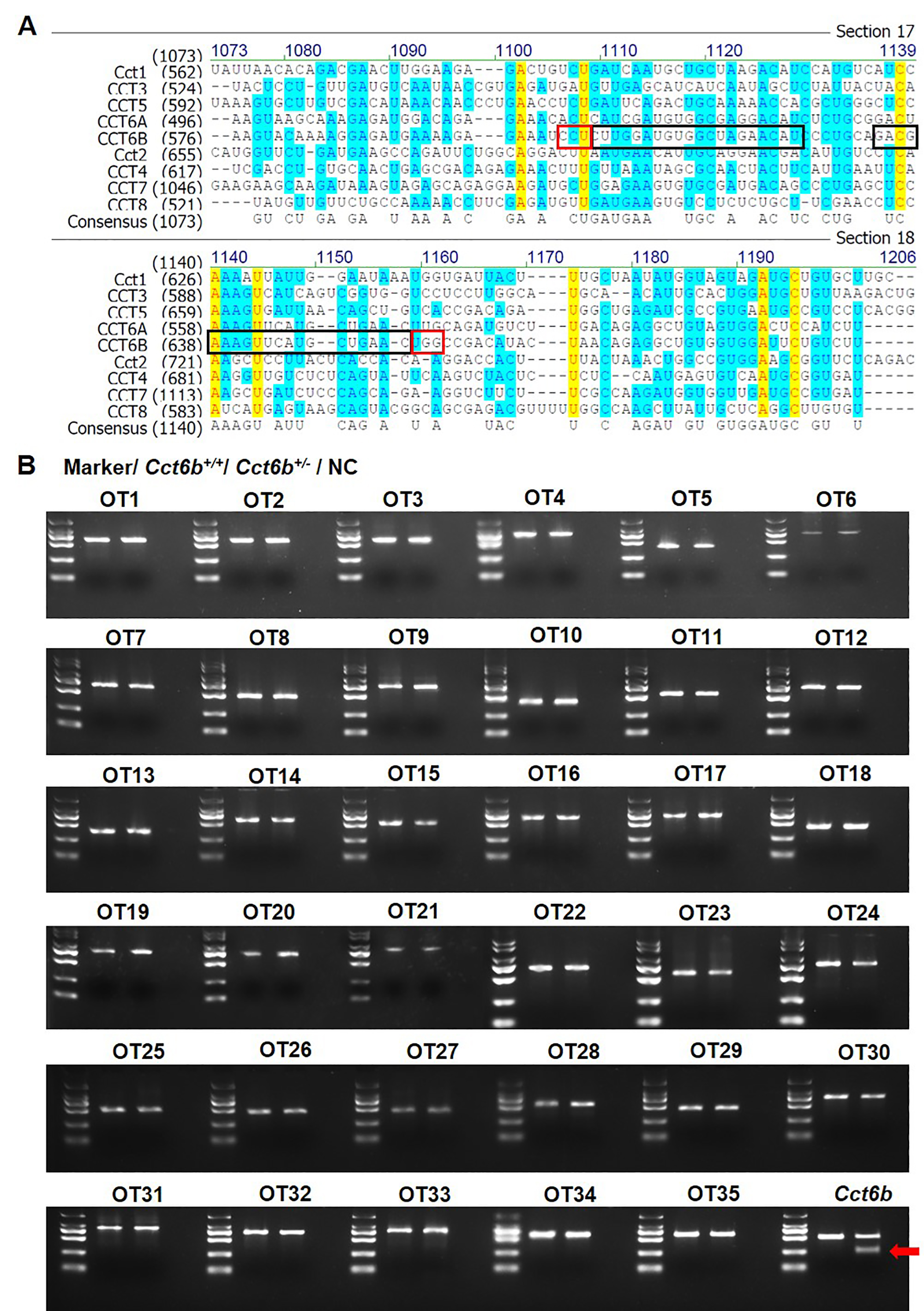

Supplement: Supplemental Information 3 — A. Sequence alignment of CCT family proteins. A black box is used to indicated the sgRNA sites, while the PAM sites are marked with a red box; B. T7 endonuclease 1 (T7E1) cleavage assay results for PCR products assessing potential off-target sites. The arrow marks the band cleaved by the T7E1. OT, off-target site. [file peerj-09-11545-s003.jpg]

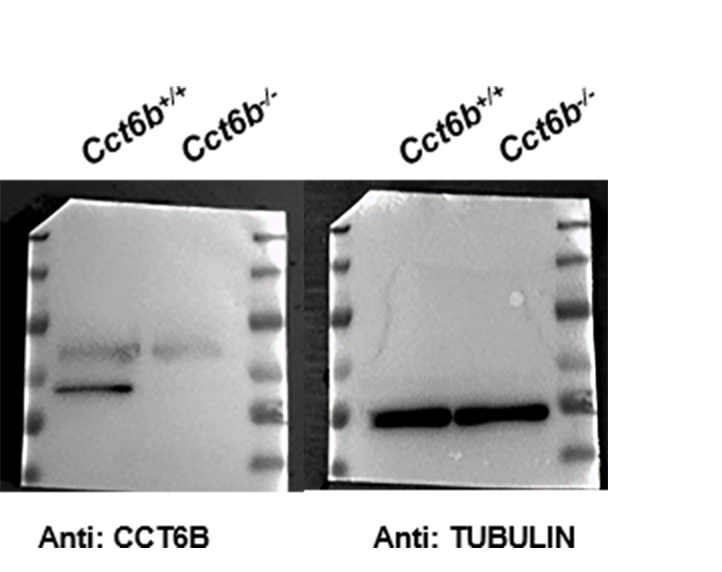

Supplement: Supplemental Information 10 [file peerj-09-11545-s010.png]
